# Analysis of tumor mutational burden: correlation of five large gene panels with whole exome sequencing

**DOI:** 10.1038/s41598-020-68394-4

**Published:** 2020-07-09

**Authors:** Carina Heydt, Jan Rehker, Roberto Pappesch, Theresa Buhl, Markus Ball, Udo Siebolts, Anja Haak, Philipp Lohneis, Reinhard Büttner, Axel M. Hillmer, Sabine Merkelbach-Bruse

**Affiliations:** 1 Institute of Pathology, Faculty of Medicine, University of Cologne, Kerpener Str. 62, 50937 Cologne, Germany; 2 Institute of Pathology, University Hospital Halle (Saale), Halle, Germany

**Keywords:** High-throughput screening, Tumour biomarkers

## Abstract

Outcome of immune checkpoint inhibition in cancer can be predicted by measuring PDL1 expression of tumor cells. Search for additional biomarkers led to tumor mutational burden (TMB) as surrogate marker for neoantigens presented. While TMB was previously determined via whole exome sequencing (WES), there have been approaches with comprehensive gene panels as well. We sequenced samples derived from formalin-fixed tumors, a *POLE* mutated cell line and standard DNA by WES and five different panels. If available, normal tissue was also exome sequenced. Sequencing data was analyzed by commercial software solutions and an in-house pipeline. A robust Pearson correlation (R = 0.9801 ± 0.0167; mean ± sd; N = 7) was determined for the different panels in a tumor paired normal setting for WES. Expanded analysis on tumor only exome sequenced samples yielded similar correlation (R = 0.9439 ± 0.0632; mean ± sd; N = 14). Remaining germline variants increased TMB in WES by 5.761 ± 1.953 (mean ± sd.; N = 7) variants per megabase (v/mb) for samples including synonymous variants and 3.883 ± 1.38 v/mb for samples without synonymous variants compared to tumor-normal paired calling results. Due to limited sample numbers in this study, additional replication is suggested for a clinical setting. Remaining germline variants in a tumor-only setting and artifacts caused by different library chemistries construction might affect the results.

## Introduction

Immune checkpoint inhibitors significantly increased survival across several tumor entities including non-small-cell lung cancer (NSCLC) and melanoma^1,2^. However, the response rate is highly variable even within a certain tumor entity, ranging from complete to no response. Thus, there is an urgent need for new predictive biomarkers to identify patients who are most likely to respond^3–5^.

Currently, two biomarkers are used to select patients: PDL1 expression as measured by immunohistochemistry is approved for companion and complementary testing prior to immunotherapy by the European Medicines Agency (EMA) and the Food and Drug Administration (FDA). In 2017, FDA also granted approval for the immunotherapy of solid tumors showing high microsatellite instability. Despite several attempts for standardization it seems that PDL1 immunohistochemistry assays alone remain insufficient as also some patients that are negative for PDL1 expression revealed response to immunotherapy^6–9^.

Retrospective studies showed the predictive ability of tumor mutational burden (TMB) to discriminate responders from non-responders across several tumor entities^10–12^. It is hypothesized that tumors with a higher mutation burden are likely to express and present more neoantigens and thereby induce a stronger immune response^13^. Supporting evidence came from the identification of other genome-related markers for response like mutations in genes related to DNA repair^14^ and deficiencies in the mismatch repair system^15^.

In previous clinical studies, whole exome sequencing (WES) was used for the measurement of TMB^16,17^. WES tumor versus normal DNA sequencing is still taken as basis for the implementation of alternative methods. Due to its higher costs, the limited tissue availability and the need of sequencing matched normal DNA, WES is of limited utility in daily clinical routine use. Recent studies and also bioinformatic approaches have shown the suitability of larger targeted gene sequencing panels for TMB assessment^18–20^.

Several commercial gene panel assays as well as reagents for laboratory developed tests (LDTs) are available^21,22^. Due to the routine use of formalin-fixed paraffin embedded (FFPE) tissue for molecular pathology diagnostics since several years preanalytical difficulties are already widely considered^23–26^. In the course of implementation of complex assays like TMB measurement other factors like composition, distribution and size of gene panels, influence of sequencing platforms, genomic coverage, bioinformatic evaluation, and definition of thresholds came into focus^27,28^. Stringent filtering criteria should be applied to exclude germline variants and artifacts related to formalin fixation, but filtering algorithms may vary between assays as well as types of mutations included for analysis. Thus, both comparison and standardization are needed and implementation in a clinical routine setting requires careful analytical validation.

In this study, we compared TMB measurements using four commercially available large targeted gene panels and one laboratory developed assay to WES. We assessed assay-to-WES correlation as well as algorithms including and excluding synonymous variants and determining germline background. Additionally, we compared variant filtering algorithms between different bioinformatic pipelines, specifically focusing upon artifacts.

## Methods

### Samples and nucleic acid extraction

Altogether 15 samples were analyzed and are listed in Supplementary information 3. 13 samples from different tumor entities and histology were selected from the registry of the Institute of Pathology of the University Hospital Cologne, Germany. The other two samples were the CW-2 cell line (sample 4) and the Horizon standard DNA (sample 9) used as internal controls for high TMB. All samples except the Horizon standard DNA were routinely formalin-fixed and paraffin-embedded (FFPE) according to local practice. 10 µm thick sections were cut from the FFPE tissue blocks and deparaffinised. Tumor areas were macrodissected from unstained slides using a marked hematoxylin–eosin (H&E) stained slide as reference.

Samples were digested overnight using proteinase K and DNA was isolated with the Maxwell® 16 FFPE Plus Tissue LEV DNA Purification Kit (Promega, Mannheim, Germany) on the Maxwell® 16 (Promega) following manufacturer’s instructions.

### Oncomine tumor mutation load assay

20 ng DNA quantified with the Qubit dsDNA HS Assay (Thermo Fisher Scientific, Waltham, MA, USA) on the Qubit 2.0 Fluorometer (Thermo Fisher Scientific) was used for library preparation with the Oncomine Tumor Mutation Load Assay (Thermo Fisher Scientific) according to manufacturer’s instructions. Library concentrations were quantified with the Ion Library TaqMan Quantification Kit (Thermo Fisher Scientific). Libraries were loaded on the Ion Chef for template preparation and chip loading using the Ion 540 Kit (Thermo Fisher Scientific), followed by sequencing on the Ion S5 XL System (Thermo Fisher Scientic).

Quality of the Ion S5 XL run was assessed with the Ion Torrent Suite 5.10 (Thermo Fisher Scientific). Data were analyzed with the Ion Reporter 5.10 (Thermo Fisher Scientific).

### NEOplus v2 RUO panel

200 ng DNA were quantified with the Qubit dsDNA HS Assay (Thermo Fisher Scientific) on the Qubit 2.0 Fluorometer (Thermo Fisher Scientific) and sheared on the Covaris E220 Focused-ultrasonicator (Woburn, MA, USA) using the 8 microTUBE–50 Strip AFA Fiber V2 following manufacturer’s instructions. The treatment time was optimized for FFPE material. The treatment settings were the following: Peak Incident Power (W): 175; Duty Factor: 10%; Cycles per Burst: 200; Treatment Time (s): 200; Temperature (°C): 7; Water Level: 6. For DNA library preparation and enrichment the NEOplus v2 RUO kit (NEO New Oncology, Cologne, Germany) was used following manufacturer’s instructions with 100 ng DNA input. Post-enriched libraries were quantified, pooled and sequenced on a NextSeq 500 (Illumina Inc., San Diego, CA, USA).

Quality of the NextSeq 500 (Illumina) sequencing runs were assessed with the Illumina Sequencing Analysis Viewer (Illumina). Sequencing data were analyzed with the NEOonsite Data Analysis RUO (version 1.4.1) and the NEO software NEOdb 2.2 (NEO New Oncology).

### TruSight oncology 500 assay

40 ng DNA were quantified with the Qubit dsDNA HS Assay (Thermo Fisher Scientific) on the Qubit 2.0 Fluorometer (Thermo Fisher Scientific) and sheared on the Covaris E220 Focused-ultrasonicator (Woburn, MA, USA) using the 8 microTUBE–50 Strip AFA Fiber V2 following manufacturer’s instructions. The treatment time was optimized for FFPE material. The treatment settings were the following: Peak Incident Power (W): 75; Duty Factor: 15%; Cycles per Burst: 500; Treatment Time (s): 360; Temperature (°C): 7; Water Level: 6. For DNA library preparation and enrichment the TruSight Oncology 500 Kit (Illumina) was used following manufacturer’s instructions. Post-enriched libraries were quantified, pooled and sequenced on a NextSeq 500 (Illumina Inc., San Diego, CA, USA).

Quality of the NextSeq 500 (Illumina) sequencing runs were assessed with the Illumina Sequencing Analysis Viewer (Illumina). Sequencing data was analyzed with the TruSight Oncology 500 Local App Version 1.3.0.39 (Illumina).

### SureSelect XT HS custom TMB and human all Exon v6 panel

Extracted DNA was quantified using the Qubit dsDNA HS Assay (Thermo Fisher Scientific) on the Qubit 2.0 Fluorometer (Thermo Fisher Scientific) and prepared for shearing according to the SureSelect XT HS Target Enrichment System Manual (Agilent, Santa Clara, CA, USA). 25–200 ng of DNA was sheared on the Covaris E220 Focused-ultrasonicator (Woburn, MA, USA) to a fragment size of 150 bp using the 8 microTUBE–50 Strip AFA Fiber V2 following manufacturer’s instructions. The treatment time was optimized for FFPE material. The treatment settings were the following: Peak Incident Power (W): 175; Duty Factor: 10%; Cycles per Burst: 200; Treatment Time (s): 200; Temperature (°C): 7; Water Level: 6.

For the custom panel, custom capture probes were designed using SureDesign (Agilent) for the target regions of 362 genes (Supplementary information 1). For library preparation SureSelect XT HS Reagent Kit (Agilent) was used following manufacturer’s instructions. In brief, pre-enriched adapter-ligated libraries were prepared. Subsequently, custom capture probes or Human all Exon v6 capture probes were hybridized to target sequences to allow for sequence enrichment using streptavidin beads. Post-enriched libraries were quantified, pooled and sequenced on a NextSeq 500 (Illumina Inc., San Diego, CA, USA).

Quality of the NextSeq 500 (Illumina) sequencing runs were assessed with the Illumina Sequencing Analysis Viewer (Illumina). FASTQ files were generated using bcl2fastq Conversion Software (Illumina). Data were further analyzed by an in-house developed pipeline based on Mutect2.

### Software pipeline for variant calling and filtering

Sequencing data was stripped from adapters by skewer^29^ followed by primary alignment via bwa^30^ and sorting with sambamba^31^. Read grouping and calling of molecular consensus reads was accomplished with fgbio (https://github.com/fulcrumgenomics/fgbio). Read groups were converted back to fastq with bedtools bamtofastq^32^ and realigned with bwa.

Variants were called with GATK 4.0.11 Mutect2^33,34^. Raw calls were annotated with snpEFF^35^ and filtered for exonic variants with SnpSift^36^. Only variants annotated as indels (insertion/deletion), SNVs (single nucleotide variants), frameshifts, affecting start/stop codons and splice site altering were allowed to pass the filter. We restricted the cutoff distance to ± 2 base pairs (bp) at the exon/intron boundary for splice sites. We also constructed a dataset that in addition contained coding synonymous variants.

Resulting vcf files were filtered by general population frequency in the non-TCGA version of the ExAC r0.3.1 database^37^, allowing only variants with minor allele frequencies < 0.01% to pass. In addition, we removed variants matching the 20,170,710 version of dbSNP150 unless they were found in the COSMIC v83 database. The threshold for variants in the tumor samples were 5 reads total as a minimum and an allelic fraction of 5% or more. At least 90% of the reads had to have a mapping quality > 1. As a measure to filter out sequencing artifacts, we used an in-house python script to screen for traces of a variant in a panel of 21 normals that had been subjected to exome sequencing with the SureSelect XT HS Exome kit (Agilent). If a variant could be detected in any normal sample, its allelic fraction (Af_normal_) was compared to the one found in the tumor (Af_tumor_). Only variants surpassing a ratio $\frac{{\mathrm{A}\mathrm{f}}_{\mathrm{tumor}}}{{\mathrm{A}\mathrm{f}}_{\mathrm{normal}}}>4$ in all 21 tumor-normal combinations were allowed to pass. If a matching normal was available, its alignment file was also added to the panel of normals to allow for a separate paired calling analysis.

The GATK 3.8 DepthOfCoverage-tool was used to determine the number of exonic basepairs with a coverage > 15 in each sample which was then used for TMB calculation.

Total coverage and average coverage for all targeted regions includes non-coding and intronic DNA on the deduplicated alignments. Total coverage was determined with bedtools coverage, average coverage was determined with GATK 3.8 DepthOfCoverage.

### QIAseq TMB panel

40 ng DNA quantified with the Qubit dsDNA HS Assay (Thermo Fisher Scientific) on the Qubit 2.0 Fluorometer (Thermo Fisher Scientific) was used for library preparation with the QIAseq Human Tumor Mutational Burden Panel (Qiagen, Hilden, Germany) according to manufacturer’s instructions. Final libraries were quantified Qubit dsDNA HS Assay (Thermo Fisher Scientific), pooled and sequenced on a NextSeq 500 (Illumina).

Quality of the NextSeq 500 (Illumina) sequencing runs were assessed with the Illumina Sequencing Analysis Viewer (Illumina). Sequencing data was analyzed with ‘Identify QIAseq DNA Somatic Variants with TMB Score (Illumina)’ v1.47 in the plugin ‘Biomedical Genomics Analysis v 1.2′ on the CLC Genomics Workbench v12.0.2 (Qiagen).

In addition to the Qiagen software, we also analyzed the data with our in-house pipeline (see description above) with minor modifications regarding the extraction of the umi (unique molecular index). Due to the different chemistry for library preparation, we also sequenced 15 normal samples independent from tumors that served as a panel of normal.

Variant annotation for filtering was done with Mutect2 FilterMutectCalls. Read_position and strand_artifact filter flags were removed for subsequent analysis. Further we employed the LearnReadOrientationModel of GATK to filter strand biases.

### Statistics

Microsoft Excel 2016, R 3.5.0 and the libraries ggplot2 and reshape2 were used for statistical calculations and graphical figures. *P* value and Bonferroni corrected p-value were calculated via a conversion of the Pearson correlation coefficient into a t-statistic. Conversion factors are the mean average TMB of the analyzed exomes divided by the mean average of the regarding panel.

### Ethics approval and consent to participate

FFPE tissue samples were obtained as part of routine clinical care under approved ethical protocols complied with the Ethics Committee of the Medical Faculty of the University of Cologne, Germany and the study was approved by the same Ethics Committee (Ethics-No. 13-091, BioMaSOTA) and written informed consent was obtained from all patients before enrollment into the study.

### Results

We sequenced 15 tumor samples derived from different tumor entities and histology and employed 5 different TMB panels, each targeting exonic regions of sizes between 1.1 and 1.3 MB (Table1). Some panels had a considerably bigger total size that included non-coding regions, e.g. for covering translocations and amplifications: TSO500 (Illumina)—1.9 MB, Oncomine Tumor Mutation Load Assay (Thermo Fisher)—1.7 MB, NEOplus v2 RUO TMB (NEO New Oncology)—2.5 MB, Qiagen TMB v3.0 (Qiagen)—1.3 MB. In addition, a custom TMB panel was designed using Agilent SureSelect XT HS chemistry, with a total size of 2.92 MB. Further, WES was conducted in this study with the Agilent SureSelect XT HS Human All Exon v6 panel. Overlap of the panel to the RefSeq coding sequences, which was used for annotation, was 35.9 MB. For the TMB gene panels, size of the coding region used for analysis is listed in Table 1. For a subset of 9 samples, there were additional matching normal tissues available. We analyzed both WES of tumor and normal tissue as pair to allow for efficient removal of germline variants. Of the 6 remaining samples without normal tissue, tumor tissue was analyzed by WES and filtered against a panel of normals.

**Table 1:** Comparison of the five TMB panels.

	Oncomine tumor mutation load assay (thermo fisher scientific)	TruSight oncology 500 assay (illumina)	NEOplus v2 RUO panel (NEO new oncology)	SureSelect XT HS custom TMB panel (agilent)	QIAseq TMB panel (qiagen)
DNA input (ng)	20	40	200/100*	10–200	40
Technology	Amplicon-based	Hybridization-based	Hybridization-based	Hybridization-based	Single primer extension
Unique molecular identifier	No	Yes	No	Yes	Yes
Genes	409	523	340	362	486
Targeted panel size (Mb)	1.7	1.9	2.5	3.1	1.3
Targeted panel size—coding region (Mb)	1.2	1.24	1.2	1.1	1.3

Comparison of features for the different gene panels; only coding regions were used in the analysis.

*for explanation, see “Methods” section.

The different software pipelines with standard configuration as supplied by the vendors were used for TMB analysis of the corresponding panels. A 5% variant allele fraction (VAF) cutoff was applied across all panels if not mentioned otherwise.

WES yielded 133.24 ± 17.86 M reads for tumors and 84.79 ± 8.03 M reads for normal samples. For the gene panels, we sequenced between 112.22 ± 41.69 M reads and 11.18 ± 2.65 M reads (Supplementary information 2).

Sample 1 was removed from the cross-panel comparison due to its low coverage in the NEOplus RUO panel (NEO New Oncology) even after resequencing. One of the WES matching normals showed tumor contamination.

This resulted in the removal of somatic variants and lead to a false, low TMB value in paired analysis when compared to tumor only results. For further analysis we excluded the contaminated normal sample. This left us with 14 samples available for comparison between the different TMB panels and WES (tumor only). Out of those, 7 had also WES matching normal samples.

### Coding synonymous variants in TMB evaluation

**Figure 1:**
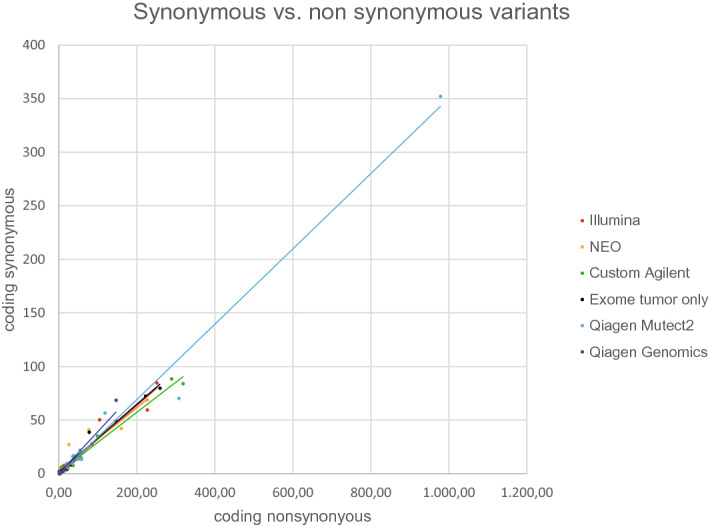
TMB values in mutations per MB of only coding non-synonymous (x-axis) and synonymous (y-axis) for bioinformatic pipelines that allowed for this differentiation. Panels: Illumina—TSO500; NEO—NEOplus v2 RUO TMB (NEO New Oncology); Custom Agilent custom—custom panel Agilent SureSelect XT HS; Exome tumor only—WES Agilent SureSelect XT HS Human All Exon v6 panel; Qiagen Mutect2—Qiagen TMB v3.0 (Qiagen) analyzed with Mutect2 in-house pipeline; Qiagen Genomics—Qiagen TMB v3.0 (Qiagen) analyzed with Qiagen Genomics Workbench 12.0.2.; Oncomine Tumor Mutation Load assay (Thermo Fisher Scientific) did not allow for differentiation of synonymous and non-synonymous variants.

Regarding the evaluation of TMB we tested the hypothesis whether coding synonymous variants, though not leading to the exposition of neoantigens, can still serve as a proxy for variants with an expected impact on protein structure. First, samples were tested for correlation of TMB values derived from non-synonymous variants and TMB values of only coding synonymous variants, when software pipelines allowed for the differentiation between coding synonymous and non-synonymous variants. This examination showed a strong correlation (R = 0.9779 ± 0.0179; mean ± sd; N = 14). Results for the different panels are shown in Fig. 1. Due to the overlap between both datasets, the association was even stronger, when all somatic variants were compared to only non-synonymous ones (R = 0,9971 ± 0,004; mean + sd; data not shown). We noticed that the ratio of called synonymous to non-synonymous variants varied between the different panels (TruSight Oncology 500 (Illumina) = 0.2815 ± 0.1611; NEOplus RUO panel (NEO New Oncology) = 0.4715 ± 0.3488; SureSelect XT HS custom TMB panel (Agilent) = 0.3856 ± 0.2122; WES (tumor only) = 0.3833 ± 0.068; QIAseq TMB panel (Qiagen) Genomics workbench = 0.6222 ± 0.0825; QIAseq TMB panel (Qiagen) Mutect2 = 0.351 ± 0.0826; Oncomine Tumor Mutation Load assay (Thermo Fisher Scientific) = N/A, only non-synonymous variants reported). This suggests that the inclusion of silent somatic variants could have a technology-dependent impact on TMB values. We therefore based further analyses on both, TMB values excluding and including synonymous variants, respectively, to assess correlation. Reference point for correlation was always the TMB of the matching exome (non synonymous variants).

### Comparison of gene panels against tumor paired normal WES

**Figure 2:**
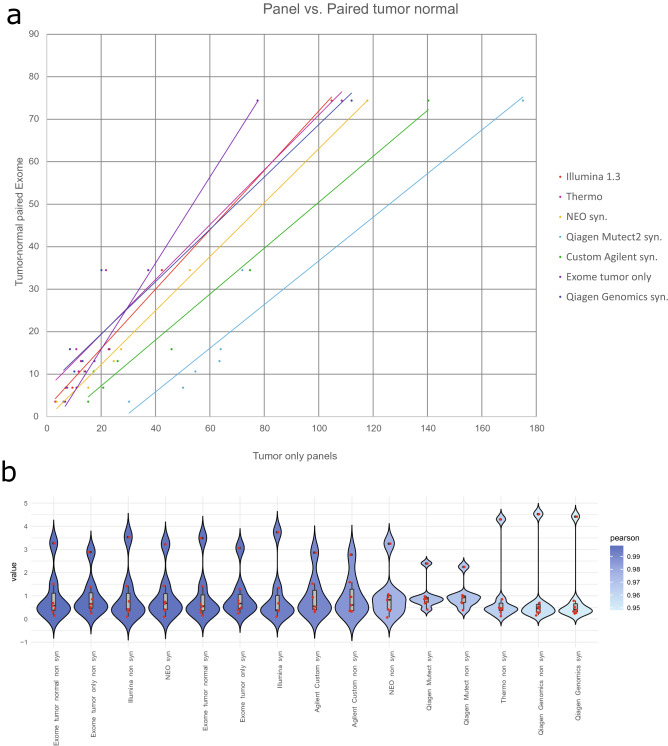
(**a**) TMB values of the panels (x-axis) compared to results from paired tumor-normal WES of non synonymous variants (y-axis). If bioinformatic pipelines delivered results including coding synonymous variants as well as excluding them, the ones with the highest correlation to the tumor-normal paired analysis are shown. Panels: Illumina—TSO500; NEO—NEOplus v2 RUO TMB (NEO New Oncology); Agilent custom—custom panel Agilent SureSelect XT HS; Exome tumor only—WES Agilent SureSelect XT HS Human All Exon v6 panel; Qiagen Mutect2—Qiagen TMB v3.0 (Qiagen) analyzed with Mutect2 in-house pipeline; Qiagen Genomics—Qiagen TMB v3.0 (Qiagen) analyzed with Qiagen Genomics Workbench 12.0.2.; Thermo—Oncomine Tumor Mutation Load assay (Thermo Fisher Scientific); syn.—Analysis includes synonymous variants. (**b**) Average normalized TMB values (y-axis) for all panels (x-axis) shown as violin plots. The red dots are the samples for the panels. The outer shape represents the density distribution and filling heat map corresponds to the Pearson correlation to exome t-n. The lower and upper hinges correspond to the first and third quartiles (the 25th and 75th percentiles) with 95% confidence interval.

We next investigated how the presence of paired normal samples as the definition of the standard for WES data affects the TMB estimates. A strong correlation (R = 0.9801 ± 0.0167; mean ± sd; N = 7) between the tumor only analysis of all the different panels and paired tumor-normal analysis of the exomes could be observed (Table 2). Correlations of all panels were significant after Bonferroni correction for multiple testing (< alpha 0.05). The different panels showed comparable distributions of TMB values relative to their averages, with the exception of the analysis of QIAseq TMB panel (Qiagen) data when analysed with our Mutect2 based pipeline displaying a smaller dynamic range (Fig. 2b).

**Table 2:** Comparison of the correlation for the different gene panels and analysis methods.

Panel	Correlation to exome tumor only	Panel	Correlation to exome tumor paired normal
Illumina 1.3	0.9950	Exome	0.9983
Agilent custom syn	0.9917	Illumina 1.3	0.9970
Illumina 1.3 syn	0.9904	NEO syn	0.9969
Agilent custom	0.9893	Exome syn	0.9968
Qiagen Genomics syn	0.9828	Illumina 1.3 syn	0.9928
Qiagen Genomics	0.9756	Agilent custom syn	0.9904
NEO	0.9582	Agilent custom	0.9867
NEO syn	0.9491	NEO	0.9760
Qiagen Mutect2	0.8877	Qiagen Mutect2 syn	0.9710
Qiagen Mutect2 syn	0.8750	Qiagen Mutect2	0.9687
Thermo non-syn	0.7886	Thermo non-syn	0.9636
		Qiagen Genomics syn	0.9549
		Qiagen Genomics	0.9484
avg	0.9439	avg	0.9801
sd	0.0632	sd	0.0167

Exome tumor only analysis for the different gene panels and analysis methods (N = 14) and exome tumor paired normal analysis for the different gene panels and analysis methods (N = 7). Panels: Illumina—TSO500; NEO—NEOplus v2 RUO TMB (NEO New Oncology); Agilent custom—custom panel Agilent SureSelect XT HS, Exome (tumor only)—WES Agilent SureSelect XT HS Human All Exon v6 panel; Qiagen Mutect2—Qiagen TMB v3.0 (Qiagen) analyzed with Mutect2 in-house pipeline; Qiagen Genomics—Qiagen TMB v3.0 (Qiagen) analyzed with Qiagen Genomics Workbench 12.0.2.; Thermo—Oncomine Tumor Mutation Load assay (Thermo Fisher Scientific); syn.–Analysis includes synonymous variants. Panels are ordered by correlation.

Based on a commonly used cutoff value of > 10 variants / MB we calculated Overall Percent Agreement (OPA), Positive Percent Agreement (PPA) and Negative Percent Agreement (NPA) of the different panels after correction by the respective conversion factors in comparison to tumor paired normal exome sequencing (Supplementary information 3). Average OPA was 88.87 ± 9.27, PPA 88.97 ± 11.79 and NPA 88.46 ± 21.07 (mean ± sd; N = 7).

Including or excluding synonymous variants had minor influence upon correlation, with the exception of the NEOplus RUO panel (NEO New Oncology), where a noticeable increase of correlation was observed (Table 2), when coding synonymous variants were included.

As the exomes were analyzed both in paired mode as well as for tumor only, the number of variants that passed filtering in both modes were compared to get an estimation of potential germline variants that had passed in tumor only analysis. We calculated a difference of 147.625 ± 53.5092 (mean ± sd.; N = 8) for non-synonymous variants and 219 ± 75.8897 variants including synonymous ones. TMB for WES tumor was increased by 5.761 ± 1.953 v/mb for samples including synonymous variants and 3.883 ± 1.38 v/mb for samples without synonymous variants compared to tumor-normal paired calling results. Suspected germline variants remaining in tumor only analysis did not correlate with the number of identified somatic variants in paired tumor normal analysis (correlation incl. synonymous = -0,2993, *p* = 0,4674; correlation excl. synonymous = − 0,292, *p* = 0,4789) and showed a smaller dynamic range than somatic variant calls.

As our own Mutect2 based pipeline allowed for adjustments for the variant allelic fraction cutoff, we lowered this value from 5 to 2% for the SureSelect XT HS custom TMB panel (Agilent) and the QIAseq TMB panel (Qiagen), to investigate how such additional low frequency variants influence TMB correlations. Interestingly, we observed opposite effects with regard to correlation between the QIAseq TMB panel (Qiagen) and the SureSelect XT HS custom TMB panel (Agilent). While the correlation to the paired tumor-normal WES for the QIAseq TMB panel (Qiagen) dropped from 0.9687 to 0.9505, it increased from 0.9866 to 0.9916 for the SureSelect XT HS custom TMB panel (Agilent) (non-synonymous variants).

We also extended the analysis to all 14 samples and evaluated the correlation between the different panels and exome tumor only TMB values. Correlation was slightly reduced to 0,9439 ± 0,0632 (mean ± sd; N = 14) (Table 2).

### Outliers in tumor only analysis

Examining the results in more detail showed deviating TMB values for sample 4 in at least two analysis pipelines, specifically our own Mutect2 based pipeline when analyzing the data obtained from the Qiagen TMB panel and the Ion Reporter 5.10 (Thermo Fisher Scientific) (Supplementary information 3). Due to the *POLE* (DNA polymerase epsilon) mutation in cell line CW-2, TMB values in sample 4 are expected to be high^38^.

Both the Ion Reporter 5.10 (Thermo Fisher Scientific) analysis as well as GATK in our own software pipeline on QIAseq TMB panel (Qiagen) data reported increased TMB values for sample 4 (Supplementary information 3). In addition, the Thermo-Fisher software reported an increased amount of variants suspected to be FFPE artifacts (data not shown).

For hybridization-based and single primer extension chemistry, it is possible to identify FFPE artifacts over a strand imbalance of the variant allelic fraction. An increased number of warnings from GATKs strand_bias filter was observed, when analyzing the QIAseq TMB panel (Qiagen) (data not shown). This might indicate an increased number of artifacts, e.g. derived from FFPE treatment during sample preparation or as an alternative hypothesis from primer artifacts that can occur during multiplex PCR. We used the experimental LearnReadOrientationModel tool of GATK to distinguish artifacts from real variants. Filtering the QIAseq TMB panel (Qiagen) data by the orientation filter increased correlation to the WES tumor only data from 0.875 to 0.9437 for variants including coding synonymous and 0.887 to 0.9412 for only non-synonymous variants, mainly by reducing the variant calls in sample 4. However, when applying the same procedure upon the smaller paired tumor-normal dataset, which does not include sample 4, correlation dropped to 0.8241/0.7330 (incl. synonymous / non-synonymous). This could be explained by much more stringent filter criteria for strand biases in this approach that might also result in unwanted filtering in samples less affected by strand imbalance prone artifacts. We also sought to determine the ratio of C:G > T:A transitions compared to the total value of variants which is a good indicator of FFPE artifacts. For the QIAseq panel, we calculated a ratio of 0.39 ± 0.11, while WES data had a ratio of 0.49 ± 0.14. For sample 4 the ratio was 0.51 (QIAseq) and 0.48 (WES).

Ratio for the QIAseq panel decreased to 0.37 ± 0.097 and in sample 4 it dropped to 0.43, when we decreased the cutoff VAF for variant filtering to 2% (data not shown).

In contrast, the CLC Genomics Workbench v12 (Qiagen) output for sample 4 in a previous version of the workflow (v. 1.35) stuck out with a much lower TMB of 22.8, as the software was using the total panel size as the denominator for the calculation of the TMB value which resulted in no normalization for the low coverage in the data of sample 4. After applying workflow 1.47 which fixed this issue during the course of our study, TMB values of the Genomics Workbench were in general complying with hybrid capture based assays.

All TMB gene panels except for WES showed reduced normalized coverage for sample 4 (Supplementary information 4). While raw sequencing output for this sample was already below average for a number of panels, deduplication further reduced the sample coverage across different panels even when the amount of input data was balanced across the samples (Supplementary information 2), which indicates a low library complexity.

## Discussion

A vast number of different factors can influence TMB values, starting with the size of the panel, tumor entity library chemistry, sequencing platform and the specific genomic regions covered by the panel^39^. Therefore, none of analyses should be considered as a ground truth. Previous results suggested that ~ 1.1 MB of exonic coding regions can be considered as a sufficient size to reliably asses TMB^40^. All tested panels fulfil this criterion. It is not clearly shown yet whether it is best to use only non-synonymous coding variants as a more direct measurement for displayed neoantigens in the tumor or if coding synonymous variants can serve as a proxy for these values^41^. Traditionally non-synonymous variants have been mostly used for TMB estimation^22^ as they have a direct influence upon protein structure and thereby neoantigen presentation of the cell. We observed a strong correlation between coding non-synonymous and coding synonymous variants across the different panels, showing that including synonymous variants might increase confidence in the determined TMB value due to higher, but still similar specific values. However, we cannot draw a final conclusion due to the limited cohort size, varying tumor entities and lack of data for patient outcome of targeted immunotherapy. Except for the NEO panel, only minor changes of correlation could be observed between analysis that included or excluded synonymous variants due to the fact that synonymous variants suffer from background noise like germline variants and artifacts in the same way that non-synonymous variants do.

Analysis of the different panels in 7 independent samples showed a robust correlation to the results from paired tumor-normal WES. It is not surprising that WES for tumor only showed the highest correlation with WES tumor-normal, as the missing normal tissue is the only difference in the analysis. However, the solutions of Illumina and NEO New Oncology (if synonymous variants were included) had similar correlation values in comparison to WES data. Comparing WES in tumor only and tumor-normal paired calling analysis allowed us to determine the overhead of variants called for tumor only. As the data was filtered against the same panel of normals, these differences can be considered as unfiltered germline variants, rather than artifacts. A correlation to the total number of somatic variants was not observed, suggesting that somatic variants and germline variants were in general properly separated. It is important to note that a relatively constant but slightly deviating number of germline variants present in a tumor-only analysis has a proportionally higher influence on the outcome of samples with lower TMB-values. When considering tumor only analyses results or alternatively cutoff values for classification into low, intermediate and high, TMB must be corrected by the average difference to make them more comparable to paired tumor-normal calling. Standard deviation for the observed difference in germline variant numbers however poses a factor of uncertainty in tumor only analysis and will play a role in individual therapeutic decisions when it shifts classification of the TMB value.

It is interesting to speculate about reasons for different levels of germline noise and how to reduce or estimate it. Ethnic background and therefore representation of variants in germline databases like dbSNP and ExAC have been shown to play a role^42^. This effect might get reduced as more, specifically non-European individuals are sequenced and their variants get incorporated into germline databases. Meanwhile ethnicity of the patient should be taken into account and germline background levels for different ethnicities need to be established for precise diagnostic TMB evaluation. Long-term developments might therefore focus on determining haplotypes rather than ethnicity of the individual to estimate the probability of germline or somatic events with more precision. While this would still lack behind sequencing of a paired normal, it could increase signal to noise ratio of the analysis.

As the comparison of TMB panel sequencing data to tumor-normal paired WES calling gave the impression of generally similar results for all TMB panels, our analysis was extended with 7 additional samples where a paired normal tissue for the WES was missing. Therefore, TMB panel data was directly compared to tumor only WES data. In one of the samples, which was isolated from the *POLE* mutated and microsatellite-instable cell line CW-2, we obtained devious results for two panels and two pipelines. We chose to keep the sample in the analysis as an example of the robustness of the different pipelines. Tumor samples of low DNA quality occur on a daily basis in pathology labs and often there is no replacement available at all. The updated version of the CLC Genomics Workbench v12 (Qiagen), though issuing a warning message regarding the low coverage still emitted TMB values similar to the hybrid capture based solutions. For time-critical clinical practice it is noteworthy, that samples with a high TMB likely will be estimated correctly, even if the sample gets heavily undersequenced.

Fixation artifacts are a complicated issue to deal with. C:G > T:A transitions have been known for a long time to appear as a predominant artifact in formalin fixed tissues^43,44^. Analysis of the Oncomine Tumor Mutation Load assay (Thermo Fisher Scientific) with the Ion Reporter 5.10 (Thermo Fisher Scientific) seeks to detect the deamination proportion by evaluating the amount of C:G > T:A variants.

While this seems to work in the majority of samples, it failed in one challenging case. Other tested solutions distinguish both strands of the DNA during amplification, and filter C > T substitutions which only occur on either + or – strand of the DNA. Both hybridization-based as well as single primer extension assays thereby rely upon a certain amount of the complementary strand to be present for this evaluation. In addition, the TruSight Oncology 500 (Illumina) hybridization-based chemistry allows for duplex calling due to its adapters, which incorporate a double stranded unique molecular index (umi). This allows for identification of the matching partner from the same fragment, if captured and sequenced over its reverse complement umi.

The QIASeq TMB assay (Qiagen), compared to the hybridization-based solutions, seems to be prone for a different kind of artifact that still needs to be determined. FFPE artifacts have been described as predominantly present in variants with lower allelic fractions^45^. On the one hand, we do observe increased strand bias. There is also decreased correlation to exome sequencing at VAF cutoff 2% while correlation in our Agilent custom hybrid capture panel increases under these circumstances. This might be a hint for more false positive variants with smaller VAF in the QIAseq data. However we do not observe the expected C:G > T:A transitions. Neither are they more prominent in QIAseq data when compared to WES nor do they increase in the lower VAF spectrum. An alternative explanation might be artifacts based on the priming site of gene specific primers during the enrichment PCR of the library preparation.

Regarding the observed strand biases, our own software pipeline yet seems to lack precision with Qiagens primer extension chemistry.

Due to the limited sample size, interchangeability between different assays should not be suggested. A switch in routine diagnosis from WES to a panel should be well prepared by analyzing a larger batch of samples of the specific tumor entity with both methods. A recent in silico study also suggested that certain tumor entities might influence panel based TMB assessment more than others^39^. Different cutoff values will need to be applied to calculate agreement between WES and the preferred test as optimal treatment outcome is associated with tumor subtype specific cutoffs^46^. One of the main questions will be which test to choose. Agreement with established methods like WES should be considered, but for the most part did not appear to be an issue in this study. Establishment of a custom panel requires additional work, but offers the benefit of screening genomic regions of interest, which might spare the user running an additional assay. On the other hand required amount and quality of DNA as well as turnaround time and ability to cope with low quality input material also play an important role in routine diagnosis.

## Conclusion

In addition to PDL1 testing, estimation of TMB has been shown to be an important biomarker for the outcome of targeted immunotherapy. As we showed in our study, available assays and software solutions are in general comparable. Switching from one assay to another therefore might only require either adjustments for cutoff values of high, intermediate and low TMB values or alternatively a direct translation in the form of a linear equation. Including coding synonymous variants in the TMB analysis did not improve correlation for the different assays/pipelines in general. While variations in germline background appeared to be manageable in our study in tumor only WES, we cannot draw a conclusion for the other assays.

We observed a complex behavior of tested solutions with regard to artifacts related to DNA fixation or sequencing, that manifest in certain basepair exchanges and strand biases. Not only seem some wet lab assays to be more prone for artifacts, their output data also provides different opportunities for error correction during downstream analysis. Our analysis showed that these artifacts need to be evaluated and addressed properly during data processing. The design and analysis of our own panel showed, that it is in fact possible to design custom solutions for assay and data processing.

## Supplementary information

Supplementary Information 1.

Supplementary Information 2.

Supplementary Information 3.

Supplementary Information 4.

## Data Availability

The datasets generated and/or analysed during the current study are available from the corresponding author on reasonable request.
